# Encapsulation of protein/DNA complexes into unilamellar liposomes via annexin-mediated membrane recruitment and sonication

**DOI:** 10.1016/j.crmeth.2025.101073

**Published:** 2025-06-09

**Authors:** Michael Burger, Finn Brigger, Valeria Mantella, Jean-Christophe Leroux

**Affiliations:** 1 Swiss Federal Institute of Technology Zurich (ETHZ), Department of Chemistry and Applied Biosciences, Institute of Pharmaceutical Sciences, Vladimir-Prelog-Weg 3, 8093 Zurich, Switzerland

**Keywords:** DNA encapsulation, protein encapsulation, liposomes, biotechnology, protein engineering, DNA delivery, synthetic biology, membrane recruitment, large unilamellar vesicle, giant unilamellar vesicle

## Abstract

This paper reports an effective protocol to encapsulate native protein/DNA complexes into unilamellar vesicles composed of natural lipids without the use of organic solvents, in physiological buffers, and at low protein/DNA concentrations. DNA compaction is achieved with the human mitochondrial transcription factor A (TFAM), which forms complexes (TFAMoplexes) when mixed with plasmid DNA (pDNA). The complexes are recruited to the surface of preformed giant unilamellar vesicles (GUVs) with the help of human annexin A4 and thereby concentrated at the membranes. This is followed by transforming the TFAMoplex-coated GUVs into small vesicles using short sonication pulses. This method results in the encapsulation of around 40% of the TFAMoplexes into unilamellar liposomes with an average hydrodynamic diameter of 121 nm. By harnessing the functions of human proteins, this approach enables the creation of complex molecular assemblies that will pave the way for a wide array of biochemical and biomedical applications.

## Highlights

• Annexin A4 enables calcium-dependent membrane recruitment of DNA-protein complexes
• Sonication of coated GUVs yields unilamellar vesicles with encapsulated TFAMoplex
• Approximately 40% of TFAMoplexes are protected within vesicles from nucleases
• The method achieves DNA/protein encapsulation under aqueous, physiological conditions

## Motivation

The fundamental blueprint of all known living organisms and viruses is encoded in DNA or RNA, which is organized, read, and replicated by specific enzymes, and typically enclosed within a lipid membrane. Efficient co-encapsulation of DNA-protein complexes into unilamellar vesicles is therefore a key step toward the development of cell-like and virus-like systems. However, existing encapsulation methods often rely on high DNA/protein concentrations or the use of organic solvents, which may compromise the integrity of native proteins. Our objective was to establish an encapsulation strategy that operates under diluted, solely aqueous conditions—offering an approach for constructing complex biological systems.

## Graphical abstract

**Figure:**
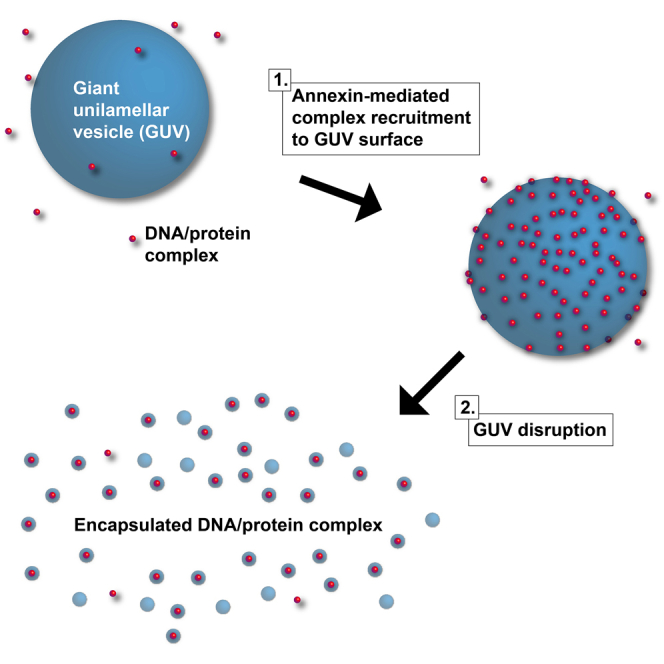

## Teaser

In this study, Burger et al. introduce a method for co-encapsulating native protein/DNA complexes into unilamellar liposomes by annexin-mediated membrane recruitment and gentle sonication. The approach offers a solvent-free, physiologically compatible route for building synthetic biological systems and advanced gene delivery platforms.

## Introduction

Liposomes are vesicles composed of one or several phospholipid bilayers that form a relatively stable compartment capable of encapsulating macromolecules.^1^ These structures can maintain specific buffer conditions and provide protection from environmental factors, such as degradative enzymes. Consequently, liposomes are used in various pharmaceutical and biotechnological applications.^2^ For instance, in gene delivery, the co-encapsulation of DNA and enzymes within unilamellar liposomes enables the creation of transfection systems that can mimic the architecture of viruses or extracellular vesicles. The incorporated proteins perform crucial functions, such as protecting the viral genome, facilitating cell entry and intracellular trafficking, or suppressing the target cell’s immune response. Reliable co-encapsulation of DNA with active proteins or enzymes could therefore open up new possibilities for designing potent, virus-mimicking transfection agents. Additionally, liposomes can provide pL-scale reaction chambers that are used to study biological processes or to generate artificial organelles within (semi)synthetic cells.^3^^,^^4^

Most strategies for DNA encapsulation rely on cationic or ionizable lipids, which effectively cluster nucleic acids through a combination of electrostatic and hydrophobic interactions, leading to the formation of lipoplexes rather than liposomes.^5^^,^^6^ These lipoplexes are typically filled with the lipid components and small aqueous compartments containing tightly compacted DNA. However, the high charge density and hydrophobic regions within lipid aggregates are unfavorable for preserving natively folded proteins, and their formation often requires organic solvents or low pH conditions. Furthermore, lipoplexes typically feature lipid monolayers on their surfaces rather than the phospholipid bilayers characteristic of liposomes. The bilayers provide the potential to incorporate transmembrane proteins and the ability to mimic cellular or vesicular surfaces. Therefore, we aim to encapsulate DNA and proteins within liposomes featuring a single (unilamellar) membrane layer and a hydrophilic core.

Several methods for encapsulating DNA into unilamellar liposomes have been reported. They typically involve precipitating DNA with organic solvents, such as ethanol, and lipids, followed by solvent removal.^7^^,^^8^ However, this is generally unsuitable for the co-encapsulation of proteins, as they can be denatured upon exposure to organic solvents. Another method involves hydrating lipid films with a highly concentrated DNA aqueous solution. The resulting multilamellar vesicles (MLVs) are then reduced in size and lamellarity by repeated extrusions through nanoporous membranes, mechanical homogenization, sonication, freeze-thaw cycles, or a combination of these techniques, ultimately producing small unilamellar vesicles (SUVs) with diameters below 100 nm or large unilamellar vesicles (LUVs) of 100–1,000 nm.^2^^,^^9^^,^^10^ The encapsulation efficiency of the lipid-film-rehydration approach is highly dependent on the molecular weight of the macromolecules and is generally unsuitable for plasmid DNA (pDNA)—due to its large size, negative charge, and susceptibility to shear forces.^11^

**Figure 1:**
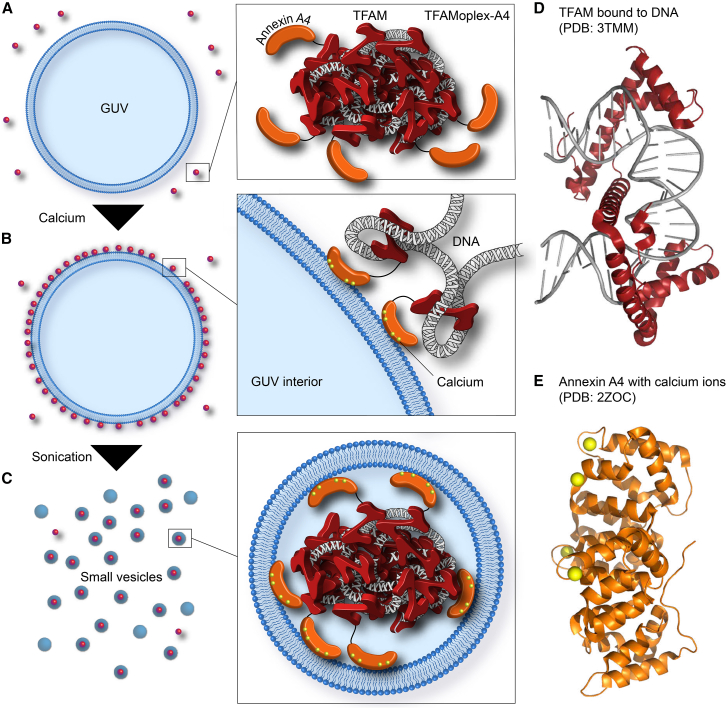
Schematic representation of the encapsulation strategy (A) TFAMoplex-A4 is added to the exterior buffer of anionic giant unilamellar vesicles (GUVs). (B) Calcium allows TFAMoplex-A4 to be recruited to the anionic GUV membranes. (C) The GUVs are transformed into large unilamellar vesicles (LUVs) through sonication pulses, thereby encapsulating TFAMoplex-A4. (D) X-ray structure of human wild-type TFAM bound to DNA (PDB: 3TMM).^22^ (E) X-ray structure of human annexin A4 (PDB: 2ZOC) with Ca^2+^ (yellow spheres).

In this study, we developed a biochemical protocol for the co-encapsulation of pDNA complexed with native proteins into LUVs (Figure 1). The proposed encapsulation strategy is based on three key principles. First, the large DNA molecules must be compacted to a size equal to or smaller than the final vesicle. This is achieved by using the human mitochondrial transcription factor A (TFAM), which forms ∼100-nm DNA/protein complexes (TFAMoplexes).^12^^,^^13^ Notably, in human mitochondria, TFAM acts as a primitive histone, compacting mitochondrial DNA and organizing transcription and genome replication.^14^^,^^15^ This biomimetic DNA compaction approach is distinct from the physicochemical methods involving charged polymers or lipids. Second, the encapsulation of macromolecules is generally most efficient when the latter interacts with the membrane, such as through charge-based interactions.^10^ Here, we achieved membrane recruitment with the help of human annexin A4, which was fused to the C terminus of TFAM and thus incorporated into the TFAMoplex (TFAMoplex-A4). We used a phosphomimetic version of annexin A4 (threonine at position 7 mutated to aspartic acid [T7D]), which is less prone to induce membrane aggregation.^16^ Annexin A4 is prevalent in both the intra- and extracellular environment and binds anionic membranes in the presence of calcium ions.^17^ It allowed the controlled recruitment of TFAMoplex-A4 to the membranes prior to vesicle formation, ensuring high DNA concentrations directly at the bilayer. Third, the recruitment membranes should be unilamellar, as in MLVs, only the outermost membrane layer would be accessible to the TFAMoplex, inevitably hampering the encapsulation efficiency. We therefore used giant unilamellar vesicles (GUVs),^18^^,^^19^ which, after coating with TFAMoplex-A4, were transformed into LUVs through short sonication pulses.^20^^,^^21^

## Results

### TFAM-annexin A4 promotes calcium-dependent DNA recruitment to membrane

**Figure 2:**
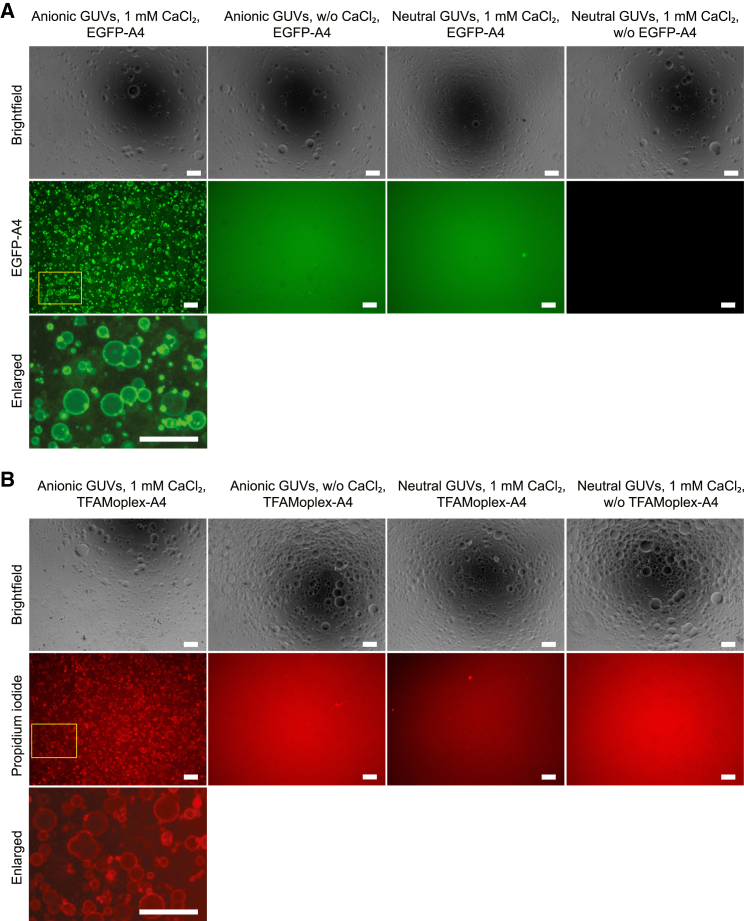
Microscopy analysis of annexin A4-dependent recruitment to the GUV surface (A) Anionic GUVs (POPC/DOPS/Chol) or neutral GUVs (POPC/Chol) were incubated with recombinant EGFP-Annexin A4 T7D in the presence or absence of 1 mM CaCl_2_. Images were captured after 15 min of incubation. Scale bars: 100 μm. (B) TFAMoplexes containing TFAM-A4 were incubated with anionic and neutral GUVs as in (A). The DNA was stained with propidium iodide. Scale bars: 100 μm. A zoom-in of the yellow-labeled region is shown in (A) and (B). All images were processed with identical brightness values. The full images showing all the tested conditions are available in Figures S1 and S2.

First, the TFAMoplex was recruited to the surface of GUVs with annexin A4 T7D. Empty GUVs were prepared using the water-in-oil emulsion-transfer method by applying an isosmotic sucrose/glucose buffer system.^18^ The GUVs were composed of 1-palmitoyl-2-oleoyl-glycero-3-phosphocholine (POPC), cholesterol (Chol), and the anionic lipid 1,2-dioleoyl-sn-glycero-3-phospho-L-serine (DOPS) in a molar ratio of 50:30:20. As a control, neutral GUVs were prepared with only POPC and Chol (70:30 mol/mol). We demonstrated that recombinant annexin A4 T7D fused to enhanced green fluorescent protein (EGFP-A4), which was added to the exterior buffer of empty GUVs, was recruited to the GUV surface within minutes but only when the GUV contained DOPS and in the presence of 1 mM CaCl_2_ (Figures 2A, S1, and S2). Without CaCl_2_ or DOPS, no surface recruitment of EGFP-A4 occurred. The size and appearance of the GUVs remained unchanged for at least 30 min. The annexin incubation time with GUVs was set at 15 min. Next, we tested the membrane recruitment of TFAMoplexes by creating the recombinant fusion protein TFAM-annexin A4 T7D (TFAM-A4). When TFAM-A4 was mixed with pDNA, complexes (TFAMoplex-A4) with a hydrodynamic diameter of 111 nm and a polydispersity index (PDI) of 0.29 were obtained (Figures S3A and S3B). In the presence of 1 mM CaCl_2_, the diameter of the TFAMoplex-A4 decreased to 97 nm (PDI = 0.20). The complexes were then added to the GUVs, both in the absence and presence of 1 mM CaCl_2_, and the DNA was stained with the fluorescent DNA intercalator propidium iodide (Figure 2B). Notably, TFAMoplex-A4 was recruited to the GUV surface in a process dependent on DOPS and CaCl_2_.

### Short sonication pulses transform GUVs into LUVs

Since the annexin A4-dependent recruitment to negatively charged GUV surfaces was successful, we next attempted to encapsulate the DNA. This was achieved using short sonication pulses with a probe sonicator. First, we aimed to ensure that the sonication process did not destroy the complexed DNA. To test this, the TFAMoplexes were subjected to increasing numbers of 1-s sonication pulses at the lowest possible amplitude. The integrity of the TFAMoplexes was subsequently assessed by agarose gel electrophoresis (Figures S3C and S3D). Each additional sonication pulse induced degradation of the DNA. After three rounds of sonication, the TFAMoplex band intensity decreased to 68% ± 10%, and after six pulses, it further declined to 39% ± 16%. We next sought to determine the number of sonication pulses required to transform GUVs into 100-nm liposomes. As for the TFAMoplexes, the GUVs were subjected to increasing numbers of 1-s pulses, and the resulting samples were analyzed by dynamic light scattering (DLS) and wide-field microscopy (Figures S3E–S3I). After five 1-s sonication pulses, most of the GUVs had disappeared, resulting in a substantial increase in small particles with few aggregates. As a compromise to minimize DNA degradation, the encapsulation was performed with 3 pulses.

### Membrane-bound TFAMoplex-A4 is encapsulated by sonication

**Figure 3:**
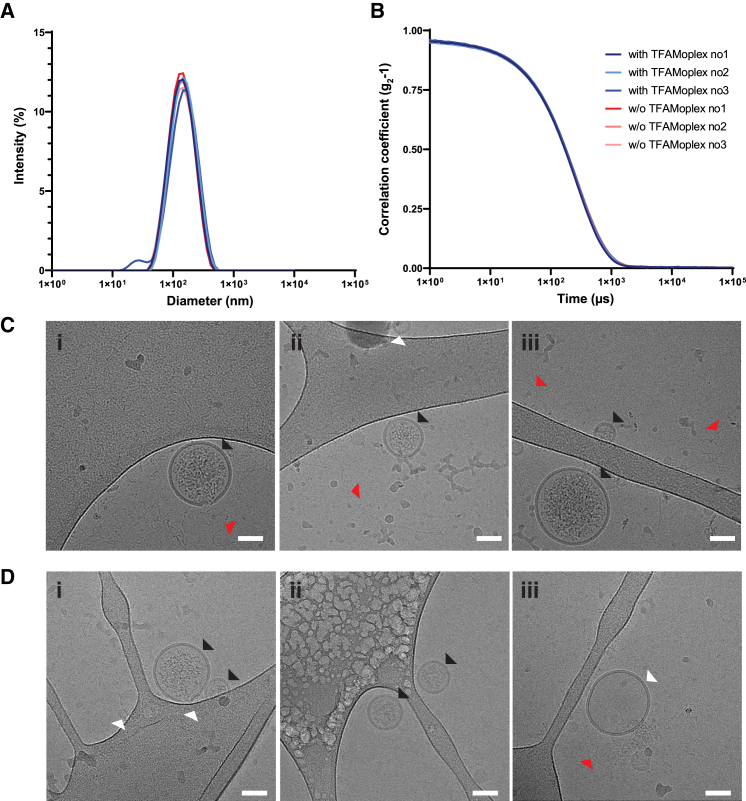
Analysis of sonicated vesicles (A) DLS measurements of vesicles after GUV sonication. Before sonication, anionic GUVs were either incubated with TFAMoplexes containing TFAM-A4 or left untreated. CaCl_2_ was added in all conditions. The sonicated samples were centrifuged, and the supernatants were analyzed by DLS. The experiment was performed independently three times, with mean values of technical triplicates displayed as curves. (B) Correlation curves corresponding to the size analysis shown in (A). (C and D) Cryo-TEM images of sonicated vesicles, either untreated (C) or treated with DNase (D). Black arrowheads indicate “filled” vesicles, white arrowheads indicate “empty” vesicles, and red arrowheads highlight free DNA. Scale bars: 50 nm. Additional cryo-TEM images are available in Figures S4 and S5. The embedded legend corresponds to (A) and (B).

To encapsulate the TFAMoplex-A4, it was first recruited to the GUV surface, followed by three 1-s sonication pulses. The remaining GUVs and lipid aggregates that can form during GUV production and sonication were removed by subsequent low-speed centrifugation. The supernatant was analyzed by DLS (Figures 3A and 3B), revealing a 121-nm peak with a PDI of 0.23. In the absence of TFAMoplex-A4, both the size of the vesicles (124 nm) and the PDI (0.25) remained nearly identical, suggesting that TFAMoplex-A4 recruitment did not significantly influence the formation of smaller vesicles during sonication. Analysis of the supernatant by cryo-transmission electron microscopy (cryo-TEM) predominantly revealed unilamellar vesicles within the expected diameter range (Figures 3C, 3D, S4, and S5). Additionally, a substantial fraction of these vesicles seemed to contain particulate matter, likely corresponding to the encapsulated TFAMoplexes. As expected, samples not treated with DNase showed free DNA that was clearly visible in the cryo-TEM images (Figures 3C and S4). After DNase treatment, extravesicular DNA was observed in only a few images, while the vesicle content remained unchanged (Figures 3D and S5).

### TFAMoplex-A4 is protected from DNase in intact LUVs

**Figure 4:**
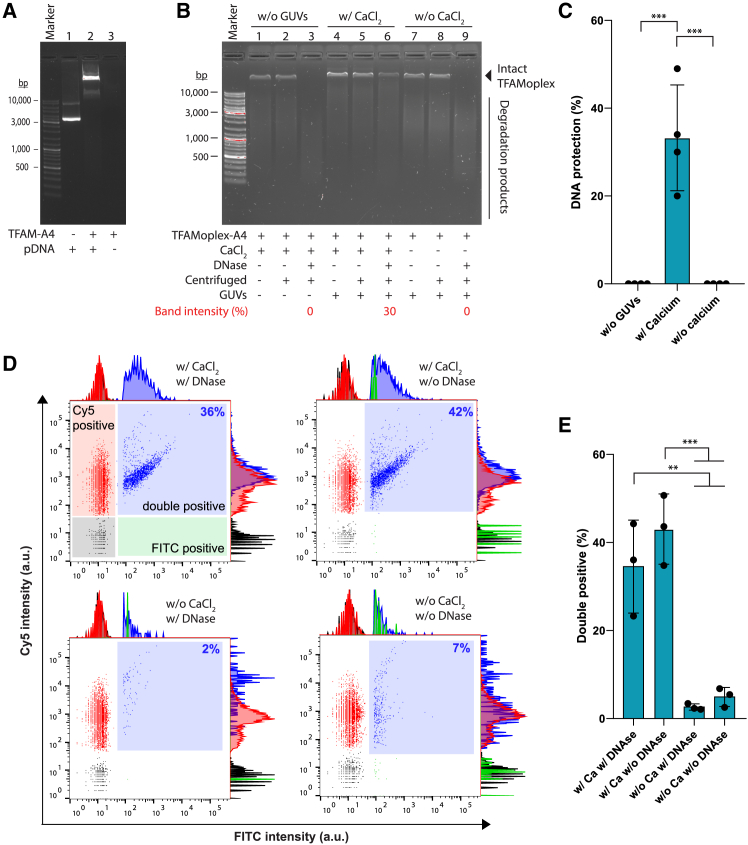
DNA encapsulation in vesicles (A) Gel mobility shift assay of TFAMoplex on a 0.8% agarose gel stained with GelRed to visualize DNA. Lane 1: “naked” pDNA; lane 2: pDNA complexed with 1 μM TFAM-A4; lane 3: TFAM-A4 without DNA. (B) DNase protection of encapsulated DNA. Encapsulation was performed without GUVs (lanes 1–3), with GUVs and Ca^2+^ (lanes 4–6), and with GUVs but without Ca^2+^ (lanes 7–9). After sonication, samples were centrifuged to remove potential aggregates and treated with DNase as indicated. The intensities of TFAMoplex DNA bands were compared between DNase-untreated and DNase-treated samples (i.e*.,* lane 1 vs. 3, lane 4 vs. 6, and lane 7 vs. 9) by densitometric analysis, with values for the displayed gel represented in red. Shown is a representative agarose gel with GelRed-stained DNA. (C) Bar plot summarizing DNA protection values derived from the analysis in (B) (mean ± SD, *n* = 4). (D and E) nanoFCM analysis of small vesicles containing Cy5-PC and FITC-labeled DNA within TFAMoplex-A4. (D) Representative bivariate dot plots of Cy5 (*x* axis) vs. FITC (*y* axis), with corresponding histograms shown alongside. The percentages of the double-positive (Cy5+/FITC+) particle populations are shown relative to the total number of recorded particles. (E) Bar plot depicting the percentages of double-positive particles in the total particle population. Bars represent mean values from three independent experiments (dots), each performed in technical triplicates (mean ± SD, *n* = 3). ∗∗*p* < 0.01 and ∗∗∗*p* < 0.001.

To determine the DNA encapsulation efficiency in LUVs, we first added DNase to the extravesicular buffer (Figures 4B and 4C). Since DNase cannot permeate intact vesicle membranes, only unencapsulated DNA, surface-bound DNA, or DNA within damaged vesicles should be degraded. Following DNase treatment, ethylenediaminetetraacetic acid (EDTA) was added to inactivate the enzyme, and the vesicles were subsequently lysed with Triton X-100. The resulting samples were analyzed by agarose gel electrophoresis with GelRed staining to visualize the DNA. The experiment revealed that 20%–50% of the DNA was protected from DNase degradation, providing an indirect estimate of the encapsulation efficiency. Notably, DNA protection was negligible when encapsulation was attempted using pre-sonicated 100-nm vesicles instead of GUVs (Figure S6). This suggests that efficient sonication-mediated encapsulation relies on large, relatively flat membrane surfaces, while small, highly curved vesicle surfaces are less effective for this process.

Finally, we confirmed DNA encapsulation using nano-flow cytometry (nanoFCM). To label the vesicle membranes, 0.25% Cy5-labeled 1,2-dioleoyl-sn-glycero-3-phosphocholine (PC-Cy5) was incorporated into the lipid mixture (POPC/DOPS/Chol/PC-Cy5), while the TFAMoplex was prepared with fluorescein isothiocyanate (FITC)-labeled pDNA (i.e., TFAMoplex-A4-FITC) (Figures 4D and 4E). Five experimental conditions were tested: vesicles prepared without TFAMoplex-A4-FITC; in the presence of TFAMoplex-A4-FITC and 1 mM Ca^2+^, with and without DNase treatment; and with TFAMoplex-A4-FITC but without Ca^2+^, with or without DNase treatment. The nanoFCM measurements revealed four distinct particle/vesicle populations: (1) double-negative vesicles that lack both Cy5-PC and FITC-DNA; (2) FITC-positive particles, which may represent naked DNA or unencapsulated TFAMoplexes; (3) Cy5-positive particles, indicating empty vesicles or lipid aggregates; and (4) the double-positive population, which likely contains vesicles that are either loaded with FITC-DNA and/or carry FITC-DNA on their surface. The DNase treatment was performed to remove free and surface-bound FITC-DNA. Notably, attempts to detect free FITC-TFAMoplexes by nanoFCM were unsuccessful. We assume that the low scattering intensity of the TFAMoplexes prevents their efficient detection by the instrument, leading to an underrepresentation of single FITC-positive events. In the absence of Ca^2+^, the double-positive population was only 5%, which further decreased to 2.5% following DNase treatment (Figure 4E). This indicates that the TFAMoplexes were neither efficiently encapsulated nor substantially associated with the external surface of the vesicles. In the samples prepared in the presence of Ca^2+^, however, the double-positive population reached an average of 43% and decreased to 35% following DNase treatment. This suggests that Ca^2+^-dependent TFAMoplex-A4 recruitment to the GUV membranes is indeed crucial for efficient encapsulation and that most of the DNA in the double-positive population is likely encapsulated.

## Discussion

The co-encapsulation of large DNA molecules with active proteins into LUVs has long been a challenging issue. In this study, we demonstrated that two critical prerequisites significantly enhance encapsulation efficiency. First, the DNA must be compacted into stable nanoparticles of appropriate size. Second, these nanoparticles must be recruited to the vesicle membranes before the final encapsulation step. With the proposed approach, these steps can be precisely controlled, relying not on charge-based interactions but rather on carefully regulated protein functions. Furthermore, the protocol was carried out in physiological buffers and at ambient temperatures, making it particularly well suited for preserving proteins in their native state. Previous studies employed the lipid-film rehydration method to encapsulate poly(ethylenimine) (PEI)-DNA nanoparticles into LUVs, achieving 0.3%–10% DNA-loaded vesicles, while most of the liposomes remained empty.^23^ Further, the authors did not report the DNA encapsulation efficiency. In contrast, significantly higher DNA entrapment efficiencies of 65%–70% were achieved with the ethanol/calcium method,^8^ which is, however, not compatible with native proteins. Additionally, encapsulation efficiencies of approximately 10% for naked DNA in POPC vesicles were achieved by freeze-thaw treatment of MLVs, followed by repeated extrusion through 100-nm membranes.^24^ However, this latter study did not confirm whether the DNA molecules remained intact throughout the procedure, nor was the unilamellar nature of the vesicles demonstrated.

GUVs were chosen as unilamellar membrane systems for surface recruitment of the TFAMoplex. GUVs offer several advantages over LUVs, including a larger, less curved surface, ease of production, simple washing due to rapid sedimentation, and reliable unilamellarity. The water-in-oil inverted emulsion method produced GUVs with a heterogeneous size distribution, which prevents the determination of a specific vesicle size’s correlation with encapsulation efficiency. In contrast, LUVs with a diameter of approximately 100 nm proved ineffective at encapsulating the TFAMoplex. This suggests that either the membrane curvature or the total surface area of the recruitment membrane plays a crucial role in successful sonication-mediated encapsulation. However, the specific impact of vesicle diameter on encapsulation efficiency remains unclear and requires further investigation.

TFAMoplex-A4 was efficiently encapsulated in vesicles (35% efficiency) and was thus protected from nuclease activity. Only small amounts of TFAMoplexes or DNA were detected on the surfaces of the final vesicles, as indirectly suggested by nanoFCM analysis, as well as visually by cryo-TEM (Figures 3C, 3D, 4E, S4, and S5). This could be attributed to annexins’ preference for binding to negatively curved or flat lipid bilayers rather than positively curved ones,^25^^,^^26^^,^^27^ as well as their ability to induce negative membrane curvature. Consequently, annexin A4 may actively facilitate membrane wrapping around the TFAMoplex during sonication-induced vesicle formation, contributing to the observed encapsulation efficiencies. However, these mechanisms require further investigation.

Protein/DNA complexes encapsulated in small vesicles hold great potential for non-viral gene delivery applications.^12^^,^^28^ However, to effectively reach the cytoplasm, these complexes must overcome both the cell or endosomal membrane and the vesicle membrane. Ideally, this challenge could be addressed by incorporating a membrane fusion system into the vesicle bilayers, facilitating the fusion of the vesicle with the target membrane and enabling the release of encapsulated material into the cytoplasm. Further research into membrane fusion machineries, particularly focusing on controlling protein incorporation into the vesicle membrane or using cell-derived membranes as a starting point for recruitment, could provide solutions to improve gene delivery.

In summary, this study advances the development of complex synthetic systems that integrate natural lipid membranes, proteins, and DNA. By utilizing the functional specificity of proteins, the process achieves a high degree of control while avoiding the use of detergents or organic solvents. This encapsulation approach presents a promising platform for future applications in biotechnology and medicine.

### Limitations of the study

The presented method for encapsulating protein/DNA complexes into unilamellar liposomes shows promising results. However, certain limitations arise from the applied GUV formation protocol, which did not allow for tight control over GUV size—and therefore membrane curvature—and restricted the range of usable lipid compositions. These factors could influence overall encapsulation efficiency and will require further optimization. Additionally, potential challenges for future *in vivo* applications, such as the immunogenicity of the protein constructs and the stability of the vesicles in biological environments, remain to be addressed.

## Resource availability

### Lead contact

Further information and requests for resources and reagents should be directed to and will be fulfilled by the lead contact, Michael Burger (michael.burger@pharma.ethz.ch).

### Materials availability

Materials used for the study are available from the lead contact upon reasonable request.

### Data and code availability

• All data supporting the findings of this study are available in the supplemental information of this article. The corresponding raw data are provided in the ETH Research Collection. DNA and protein sequences are provided in the supplemental information section.
• This paper does not report original code.
• Any additional information required to reanalyze the data reported in this paper is available from the lead contact upon request.

## Acknowledgments

We acknowledge the Scientific Center for Optical and Electron Microscopy (ScopeM) at ETH Zurich, especially Stephan Handschin, for their support with the confocal and electron microscopy studies. Further, we thank Professor Hiroaki Suzuki for helpful discussions on the GUV formation protocols. This project has received funding from the 10.13039/501100000781European Research Council (ERC) under the European Union’s Horizon 2020 research and innovation program (grant agreement no. 884505).

## Author contributions

M.B. conceived the encapsulation strategy, performed most of the experiments, and wrote the manuscript. F.B. performed the NanoFCM experiments and analyzed the data. V.M. set up the cryo-TEM imaging protocol. J.-C.L. was involved in supervision and data analysis. All authors edited and proofread the manuscript.

## Declaration of interests

The authors declare no competing interests.

## Declaration of generative AI and AI-assisted technologies in the writing process

During the preparation of this work, the authors used ChatGTP for proofreading. After using this tool, the authors reviewed and edited the content as needed and take full responsibility for the content of the publication.

## STAR★Methods

### Key resources table

**Table:** 

REAGENT or RESOURCE	SOURCE	IDENTIFIER
**Bacterial and virus strains**		

*E. coli* (DE3)BL21 pLysS	from Promega AG	L1195

**Chemicals, peptides, and recombinant proteins**		

Poly(ethyleneimine) (PEI)	Sigma-Aldrich GmbH	9002-98-6
Coomassie brilliant blue G-250	Sigma-Aldrich GmbH	20279
Kanamycin sulfate	Sigma-Aldrich GmbH	25389-94-0
Isopropyl β-D-1-thiogalactopyranoside (IPTG)	Sigma-Aldrich GmbH	B1900-500
D-glucose	Sigma-Aldrich GmbH	50-99-7
4-(2-hydroxyethyl)-1-piperazineethanesulfonic acid (HEPES)	Sigma-Aldrich GmbH	7365-45-9
Halt™ Protease and Phosphatase Inhibitor Cocktail, EDTA-free (100X)	Sigma-Aldrich GmbH	78441
Potassium acetate (KAc)	Sigma-Aldrich GmbH	127-08-2
Mineral oil (BioUltra)	Sigma-Aldrich GmbH	69794
Sucrose	Sigma-Aldrich GmbH	15503022
Chloroform	Sigma-Aldrich GmbH	67-66-3
Calcium chloride (CaCl_2_)	Sigma-Aldrich GmbH	10035-04-8
Sodium hydroxide (NaOH)	Sigma-Aldrich GmbH	1310-73-2
Glycerol	Sigma-Aldrich GmbH	032450.AE
Imidazole	Sigma-Aldrich GmbH	56750
Heparin-agarose	Sigma-Aldrich GmbH	H6508
Triton X-100	Sigma-Aldrich GmbH	X100PC
Cholesterol	Sigma-Aldrich GmbH	C8667
Potassium chloride (KCl)	Sigma-Aldrich GmbH	204099
Magnesium chloride (MgCl_2_)	Sigma-Aldrich GmbH	M8266
Tris base	Sigma-Aldrich GmbH	252859
Lysozyme	AppliChem GmbH	A3711,0010
50 x TAE buffer	AppliChem GmbH	A1691
EDTA	AppliChem GmbH	A2937
Human albumin	Sigma-Aldrich GmbH	A3782
FastDigest DNA restriction enzymes	Thermo Fisher Scientific	N/A
Nickle- nitriloacetic acid (Ni-NTA) agarose	Qiagen	30410
Phosphate buffered saline (PBS)	Thermo Fisher Scientific	10010023
UltraPure Low Melting Point Agarose	Thermo Fisher Scientific	16520100
Acetic acid	Thermo Fisher Scientific	9526–33
Methanol	Thermo Fisher Scientific	67-56-1
GelRed® Nucleic Acid Gel Stain	Biotium	N/A
Syringe filters (0.22 μm)	Sarstedt	83.1826.001
POPC	Avanti Research	850457
DOPS	Avanti Research	840035
18:1 Cy5-PC	Avanti Research	850483
Chloramphenicol	ABCR GmbH	56-75-7
Luria-Broth medium (LB)	Lab logistics group GmbH	6.271 111
TFAM-A4 T7D	This study	N/A
EGFP-A4 T7D	This study	N/A

**Recombinant DNA**		

pET His6 TEV LIC cloning vector (1B)	Addgene	Plasmid #29653
DNA fragment Annexin A4 T7D	GeneArt Thermo Fisher Scientific	see Table S1
DNA fragment EGFP	GeneArt Thermo Fisher Scientific	see Table S1
DNA fragment TFAM	GeneArt Thermo Fisher Scientific	see Table S1

**Software and algorithms**		

NanoFCM Professional Suite version 2.3	NanoFCM Co., Ltd.	https://www.nanofcm.com/
FlowJo version 10.9.0	Biosciences	https://flowjo.com/
Prism version 8.0.0	GraphPad Software, Inc.	https://www.graphpad.com/

### Experimental model and study participant details

#### Bacterial cell lines

*E. coli* (DE3)BL21 pLysS. The bacteria were cultured in LB medium supplemented with the appropriate antibiotics at 37°C (for overnight cultures) or 30°C (for protein production) and shaking at 210 rpm.

### Method details

#### Cloning TFAM-A4 and EGFP-A4

The DNA sequences encoding human annexin A4 T7D, TFAM, and EGFP were synthesized by GeneArt (Thermo Fisher Scientific) and cloned into the pET-28b vector using standard molecular cloning techniques. For TFAM and EGFP, the sequences were inserted using the restriction sites PstI and BamHI, while BamHI and XhoI were used for annexin A4. The DNA sequences of the expression plasmids are provided in Table S1. The integrity and accuracy of all constructs were confirmed by Sanger sequencing (Microsynth AG, Balgach, Switzerland) across the coding regions.

#### Recombinant TFAM-A4 purification

A saturated overnight culture of *E. coli* BL21(DE3)pLysS in LB medium supplemented with kanamycin (50 μg/mL) and chloramphenicol (30 μg/mL) was diluted 1:100 (v/v) into 700 mL of fresh LB medium containing kanamycin, chloramphenicol, and 0.2% (w/v) glucose in a 2-L baffled Erlenmeyer flask. The culture was incubated at 37°C with shaking at 210 rpm in an orbital shaker (Multitron, Infors-HT, Bottmingen, Switzerland) until the optical density at 600 nm (OD600, microplate reader Spark, Tecan, Mannedorf, Switzerland) reached 0.5. Protein expression was induced by adding IPTG to a final concentration of 0.4 mM, and the culture was further incubated at 30°C for 4–5 h.

Cells were harvested by centrifugation at 5000 × g for 10 min at 4°C (Sorvall LYNX 6000 centrifuge, Thermo Scientific). The resulting pellet was stored at −20°C overnight. The next day, the bacterial pellet was resuspended in ice-cold Buffer A (25 mM HEPES, pH 7.4, 500 mM KCl) containing 1 mg/mL lysozyme, 1% (w/v) Triton X-100, and protease inhibitor cocktail (1:100 v/v). Cells were lysed on ice by sonication (FB705 sonicator, Thermo Fisher Scientific) using a total pulse time of 3 min (5 s pulses, 10 s breaks). To remove bacterial DNA contamination, 0.1% (w/v) PEI was added, and cell debris were removed by centrifugation at 30,000 × g for 30 min at 4°C.

Imidazole was added to the supernatant to a final concentration of 10 mM, and the solution was filtered through a 0.22-μm syringe filter. The filtrate was loaded onto a Ni-NTA column (0.5–1 mL beads) equilibrated with Buffer B (25 mM HEPES, pH 7.4, 500 mM KCl, 10 mM imidazole). The column was washed with at least 10 column volumes of Buffer B followed by 5 column volumes of Buffer C (25 mM HEPES, pH 7.4, 500 mM KCl, 20 mM imidazole). Proteins were eluted with Buffer D (25 mM HEPES, pH 7.4, 500 mM KCl, 200 mM imidazole) and collected in 2 mL fractions.

To further purify TFAM-A4 and remove residual bacterial DNA, the eluate was diluted 1:4 with cold ddH_2_O and loaded onto a heparin-agarose resin (500 μL beads) equilibrated with PBS. After washing with 10 mL PBS, the protein was eluted with PBS containing 1 M KCl. A buffer exchange into Buffer E (25 mM HEPES, pH 7.4, 500 mM KCl) was performed using 3000 MWCO Amicon Ultra centrifugal filters (Sigma Aldrich, St. Louis, MO) by centrifugation at 4000 × g at 4°C (Heraeus Megafuge 16R centrifuge, Thermo Fisher Scientific). The purified protein was aliquoted, and its concentration was estimated by spectrophotometry at 280 nm (NanoPhotometer Pearl, Implen GmbH, Munich, Germany). Protein purity was assessed by SDS-PAGE (Mini-PROTEAN TGX Stain-Free Precast Gels, BioRad, Hercules, CA; Figure S6D) with Coomassie staining (0.1% Coomassie blue (w/v), 10% acetic acid (v/v), 30% methanol (v/v) in ddH_2_O), followed by densitometric analysis.

#### Gel mobility shift assay

TFAM activity was assessed using a gel mobility shift assay as described previously.^12^ A 0.8% agarose gel was prepared in 1× TAE buffer containing GelRed dye (50 μL/L). Three samples (final volume 10 μL each) were prepared in PBS: sample 1 contained 100 ng pDNA; sample 2 contained 100 ng pDNA mixed with 1 μM TFAM-A4; and sample 3 contained only 1 μM TFAM-A4. Samples were incubated for 20 min at room temperature, followed by the addition of 2 μL gel loading buffer (FastDigest Green Buffer (10X), Thermo Fisher Scientific). The samples were loaded onto the agarose gel, and electrophoresis was performed in 1× TAE buffer at 100 mA for 40–60 min. The gel was visualized using a ChemiDoc MP Gel reader (BioRad).

#### Preparation of GUVs

GUVs were produced based on previously published protocols with some modifications.^19^ Lipids dissolved in chloroform were mixed in the desired ratios in a flat-bottom glass vial to achieve a final concentration of 0.8 mM in mineral oil. The chloroform was evaporated under a nitrogen stream, and the lipid film was dried under vacuum for at least 1 h. Subsequently, 12 mL of mineral oil was added, and the solution was sonicated for 30 min at 40°C in a water bath sonicator (Ultrasonic Cleaner, Labmaterial GmbH, Egnach, Switzerland). Samples were stored in a desiccator under vacuum at room temperature and used within 2 weeks.

For GUV formation, 300 μL of exterior buffer (25 mM HEPES, pH 7.4, 50 mM KCl, 200 mM D-glucose) containing 300 μg human albumin was added to a 1.5 mL tube (tube 1) and covered with 100 μL lipid/oil mixture. Simultaneously, 350 μL lipid/oil mixture was transferred into a separate 2 mL tube (tube 2). Tube 1 was incubated at room temperature for 20 min to allow lipid monolayer formation at the buffer/oil interface. Then, 15 μL interior buffer (25 mM HEPES, pH 7.4, 50 mM KCl, 200 mM sucrose) was added to tube 2, and the emulsion was formed by pulling tube 2 repeatedly over a tube rack. The emulsion was gently transferred to the oil phase in tube 1, which was centrifuged at 18,000 × g for 3 min at room temperature. The GUVs were collected from the aqueous phase in tube 1 by puncturing the bottom of the tube with a needle (19 G, 1.10 × 40 mm, B. Braun Medical AG, Sempach, Switzerland). The aqueous phase was transferred to a clean 1 mL tube (tube 3) and centrifuged at 18,000 × g for 2 min to pellet the GUVs. The supernatant was carefully removed, leaving the GUV pellet covered with buffer. The pellet was gently resuspended by tapping the tube, yielding a concentrated, slightly milky GUV suspension.

#### TFAMoplex formation

TFAMoplexes were formed by gently mixing 1 μM TFAM-A4 protein and 500 ng plasmid DNA (pEGFP, 6.1 kbp) in a total volume of 50 μL exterior buffer. The mixture was incubated for 20 min at room temperature.

#### Membrane recruitment

TFAMplexes (50 μL) were added to 50 μL GUVs, followed by CaCl_2_ to a final concentration of 1 mM (11 μL of 10 mM CaCl_2_ stock solution in exterior buffer). The samples were incubated for 15 min to allow association of the TFAMoplex with the GUV membranes. Then the sample was diluted with exterior buffer (no albumin) to a total volume of 300 μL.

#### Vesicle sonication

GUVs were sonicated using an FB705 sonicator with probe 418-A (Thermo Fisher Scientific) with 3 × 1-s pulses (or as indicated) at an amplitude of 1, in a total sample volume of 0.3 mL. Each pulse delivered an energy of 4–6 J. To prevent sample heating, the tube was placed in a water bath at room temperature during sonication. The sample was then centrifuged at 5000 × g for 5 min at room temperature to remove aggregates and residual GUVs. The supernatant, containing the final vesicles, was collected for subsequent experiments.

#### Microscopy

Microscopy experiments were performed using a Leica DMI6000B epifluorescence microscope (Leica Microsystems, Wetzlar, Germany). To analyze protein or TFAMoplex recruitment to the GUV surface, 50 μL of GUV suspension were added to a well in a 96-well plate (TPP Techno Plastic Products AG, Trasadingen, Switzerland). Proteins or TFAMoplexes were added to the well, followed by the addition of exterior buffer and calcium (final concentration: 1 mM) to a total volume of 100 μL. GUVs were sedimented prior to imaging by centrifugation at 200 × g for 30 s at room temperature. Imaging was conducted at room temperature.

#### DNA degradation assay by sonication

TFAMoplexes were prepared as previously described in a total volume of 100 μL exterior buffer. Following TFAMoplex formation, 1 mM CaCl_2_ was added, and the mixture was incubated for 15 min at room temperature. The sample was then diluted to a final volume of 400 μL with exterior buffer and subjected to sonication with 1-s pulses at an amplitude of 1. After each sonication step, 20 μL of the sample were collected for analysis.

For each aliquot, 3 μL of gel loading buffer (FastDigest Green Buffer (10X), Thermo Fisher Scientific) were added, and 20 μL of the prepared sample were loaded onto a 0.8% (w/v) agarose gel containing GelRed DNA dye. Gel electrophoresis was performed in TAE buffer at 110 mA for approximately 40 min at room temperature. Gels were imaged using a ChemiDoc imager, and densitometric analysis was performed to quantify the extent of TFAMoplex degradation.

#### Dynamic light scattering (DLS)

Dynamic light scattering (DLS) measurements were performed using a Zetasizer Pro (Malvern Instruments Ltd., Malvern, Worcestershire, UK). To measure the hydrodynamic diameter based on light scattering intensity, the TFAMoplexes were prepared as described above and diluted to a final volume of 100 μL in exterior buffer. From this dilution, 80 μL were transferred into a micro UV cuvette (BrandTech Scientific, Essex, CT) for analysis. Triplicate measurements were recorded at 25°C with a 30-s equilibration time, using the multiple narrow analysis model. The triplicate values were averaged.

Vesicle samples were analyzed using the same protocol without dilution. Each experiment was repeated in at least three independent replicates.

#### NanoFCM

The fluoresceine-labeled TFAMoplex-A4 was formed as described above but with FITC-pDNA (2,600 bp plasmid, Label IT Plasmid Delivery Control, Mirus Bio, Madison, WI). The vesicles were formed as described above, with 0.25% (n/n) of 18:1 Cy5-PC included in the lipid mix. Samples were analyzed using the Flow NanoAnalyzer N30 (NanoFCM Co., Ltd, Nottingham, UK). Events were recorded for 1 min on single-photon counting avalanche photodiode detectors (SPCM APDs) across three channels: 488/10 (trigger channel), 525/40, and 670/30. The sampling pressure was maintained at 1.0 kPa using an air-based pressure module. The channels were aligned using fluorescent 250 nm silica QC beads and size calibration was performed with the S16M-Exo silica nanosphere cocktail (both NanoFCM Co., Ltd.). The 488-nm blue laser was set to 10/50 mW and the 638-nm red laser was set to 20/100 mW, with an SS decay of 10%. Prior to analysis, samples were diluted with exterior buffer (25 mM HEPES, pH 7.4, 50 mM KCl, 200 mM D-glucose) to achieve a final event range of 1500–5500. Control experiments included blank measurements with buffer only and unloaded Cy5-labeled vesicles. Data was analyzed using the NanoFCM Professional Suite software (version 2.3, NanoFCM Co., Ltd.). Further analysis and data visualization was performed using FlowJo software (version 10.9.0, BD Biosciences, Franklin Lakes, NJ).

#### Cryo-TEM

The cryo-TEM samples were prepared on lacey carbon EM grids (Au 300 mesh, 150 μm, Byolist Scientific, Hatfield, PA) glow-discharged using a Pelco EasiGlow system at 25 mA for 30 s to enhance hydrophilicity. A 3.4 μL aliquot of the sample solution was then applied to the carbon side of the grid and incubated for 20 min in a humidified chamber, with the microscopy grid positioned on a glass slide inside a plastic box lined with water-soaked wipes. This extended adsorption step was specifically carried out for the preparation of negatively stained grids at room temperature. For cryo-TEM imaging, the grids were plunge-frozen into a precooled liquid ethane: propane mixture using a Vitrobot Mark IV (FEI, Hillsboro, OR), maintained at 22°C.

Images were acquired on a Titan Krios FEG (Thermo Fisher Scientific) operating at 300 kV. The imaging setup included a Falcon III 4k × 4k direct electron detector (Thermo Fisher Scientific), a Ceta 16M 4k × 4k CMOS detector (Thermo Fisher Scientific), and a Gatan K2 detector with a Quantum LS energy filter (Gatan, Pleasanton, CA).

### Quantification and statistical analysis

The statistical analysis was conducted using the GraphPad Prism software, version 8.0.0. The data are represented as mean with standard deviation (SD) of at least 3 independent experiments. Significance was assessed by one-way ANOVA in combination with Tukey’s multiple comparisons test. The number of biological and technical replicates performed in each experiment, along with the corresponding *p*-value intervals, are indicated in the figure captions. *p*-values below 0.05 were considered significant. No data points were excluded from the statistical analysis. Both the cryo-TEM and NanoFCM studies were performed under blinded conditions, with the experimenter unaware of the sample identity.

## Supplemental information

Document S1. Figures S1–S6 and Table S1

Document S2. Article plus supplemental information
