# Supplementary material for: Encapsulation of protein/DNA complexes into unilamellar liposomes via annexin-mediated membrane recruitment and sonication
Source: Cell Rep Methods. 2025 Jun 9;5(6):101073. doi: 10.1016/j.crmeth.2025.101073 (PMC12272240; doi:10.1016/j.crmeth.2025.101073)
Supplement: Document S1. Figures S1–S6 and Table S1 [file mmc1.pdf]

Cell Reports Methods, Volume *5*

Supplemental information

Encapsulation of protein/DNA complexes into unilamellar liposomes via annexin-mediated membrane recruitment and sonication

Michael Burger, Finn Brigger, Valeria Mantella, and Jean-Christophe Leroux

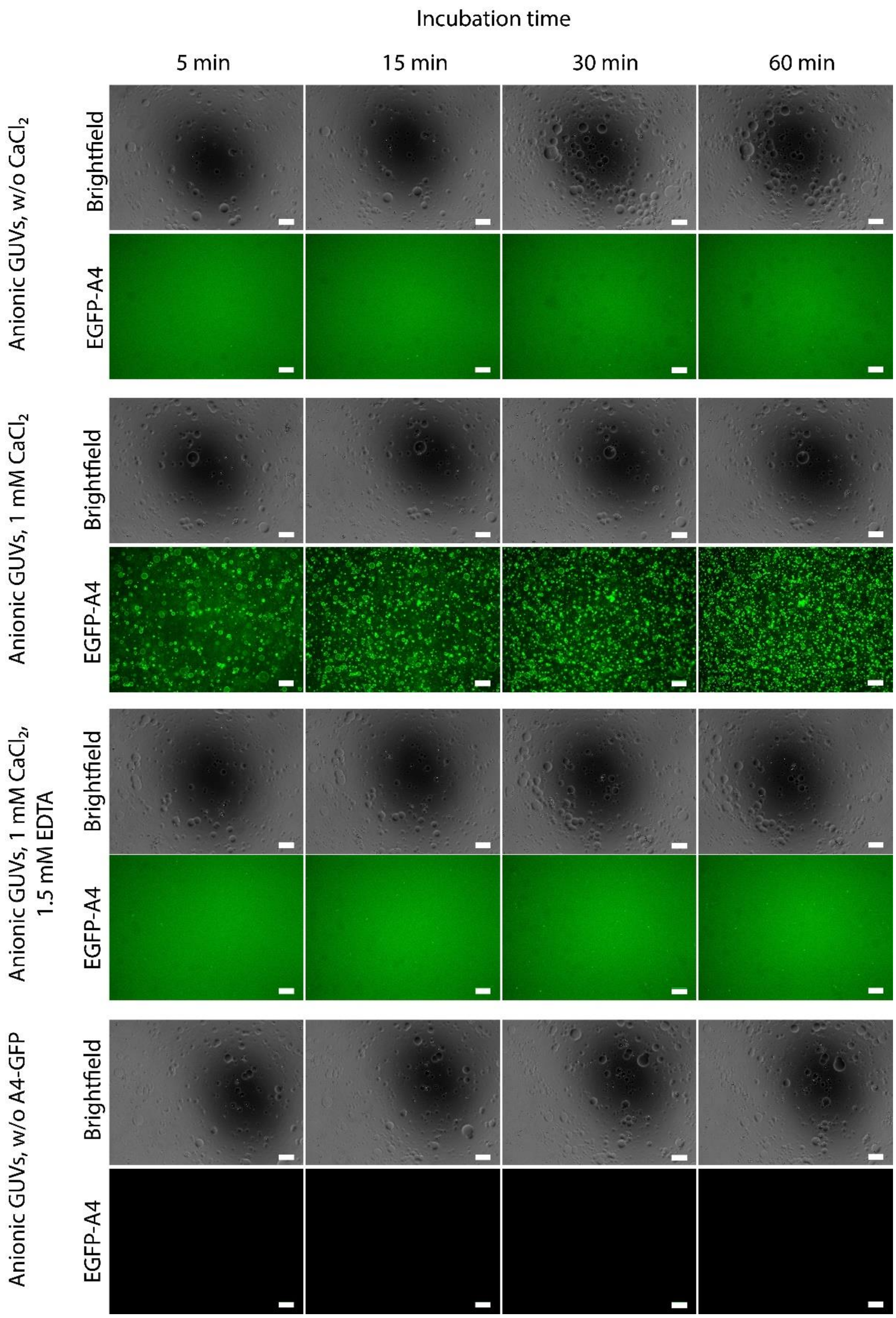

Supplementary Figure S1 – Fluorescence microscopy analysis of EGFP-A4 recruitment to the GUV surface. Anionic GUVs (50% POPC, 20% DOPS, 30% cholesterol) were incubated with EGFP-A4 to evaluate its recruitment to the membrane surface under varying conditions and time periods. The images show the same sample at different time periods. Recruitment was assessed in the presence or absence of calcium ions, with EDTA used as a

negative control to chelate calcium. The panels ‘anionic GUVs, 1 mM $CaCl_2$, 15 min’ and ‘anionic GUVs, w/o $CaCl_2$, 15 min’ are also displayed in Figure 2a in the main text. Scale bars = 100 µm. Related to Figure 2.

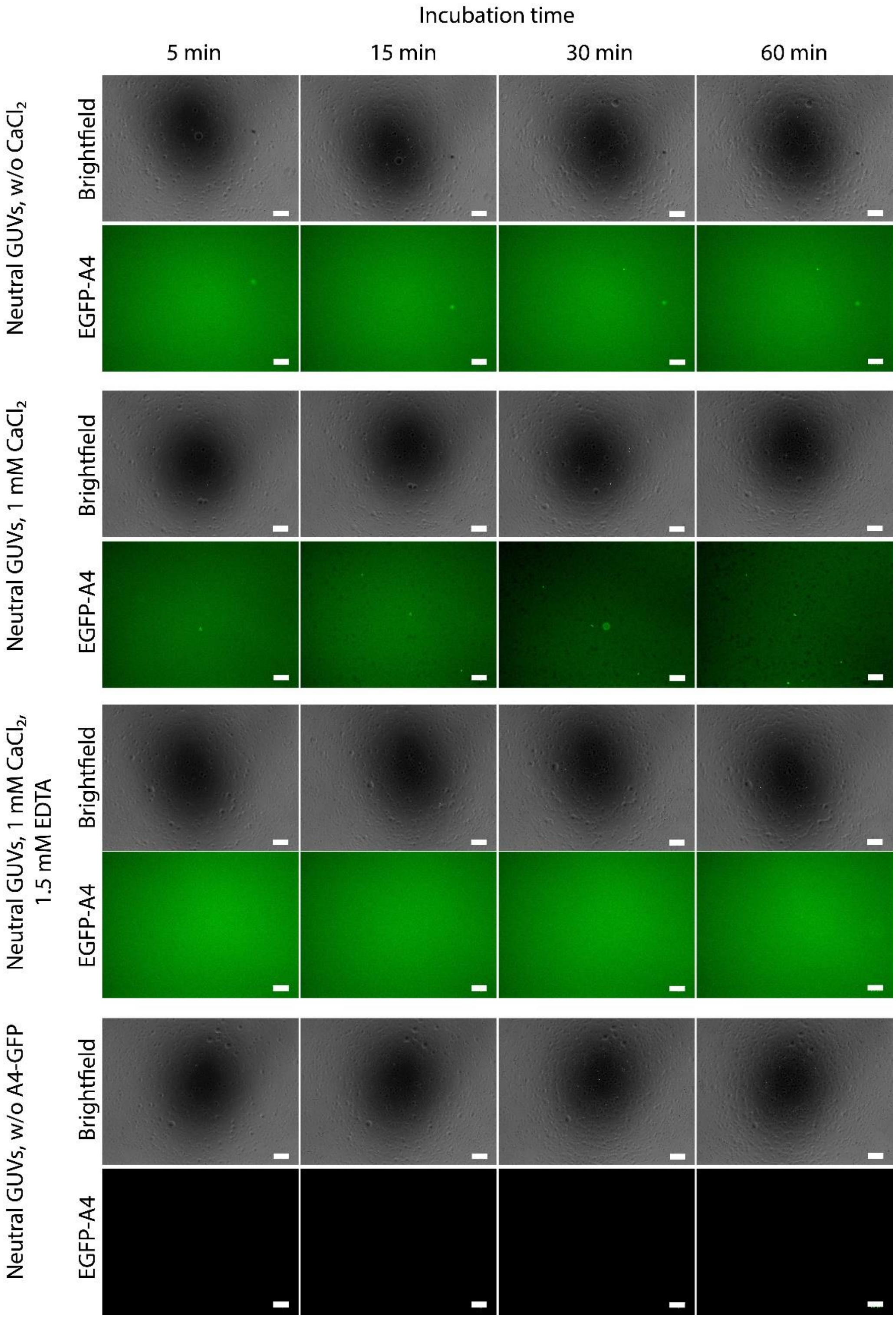

Supplementary Figure S2 – Fluorescence microscopy analysis of EGFP-A4 recruitment to neutral GUV surfaces. Neutral GUVs (70% POPC, 30% cholesterol) were incubated with EGFP-A4 to evaluate its recruitment to the membrane surface under varying conditions and time periods. The images show the same sample at different time periods. Recruitment was assessed in the presence or absence of calcium ions, with EDTA used as a negative control

to chelate calcium. The panels ‘neutral GUVs, 1 mM $CaCl_2$, 15 min’ and ‘neutral GUVs, w/o $CaCl_2$, 15 min’ are also displayed in Figure 2a in the main text. Scale bars = 100 µm. Related to Figure 2.

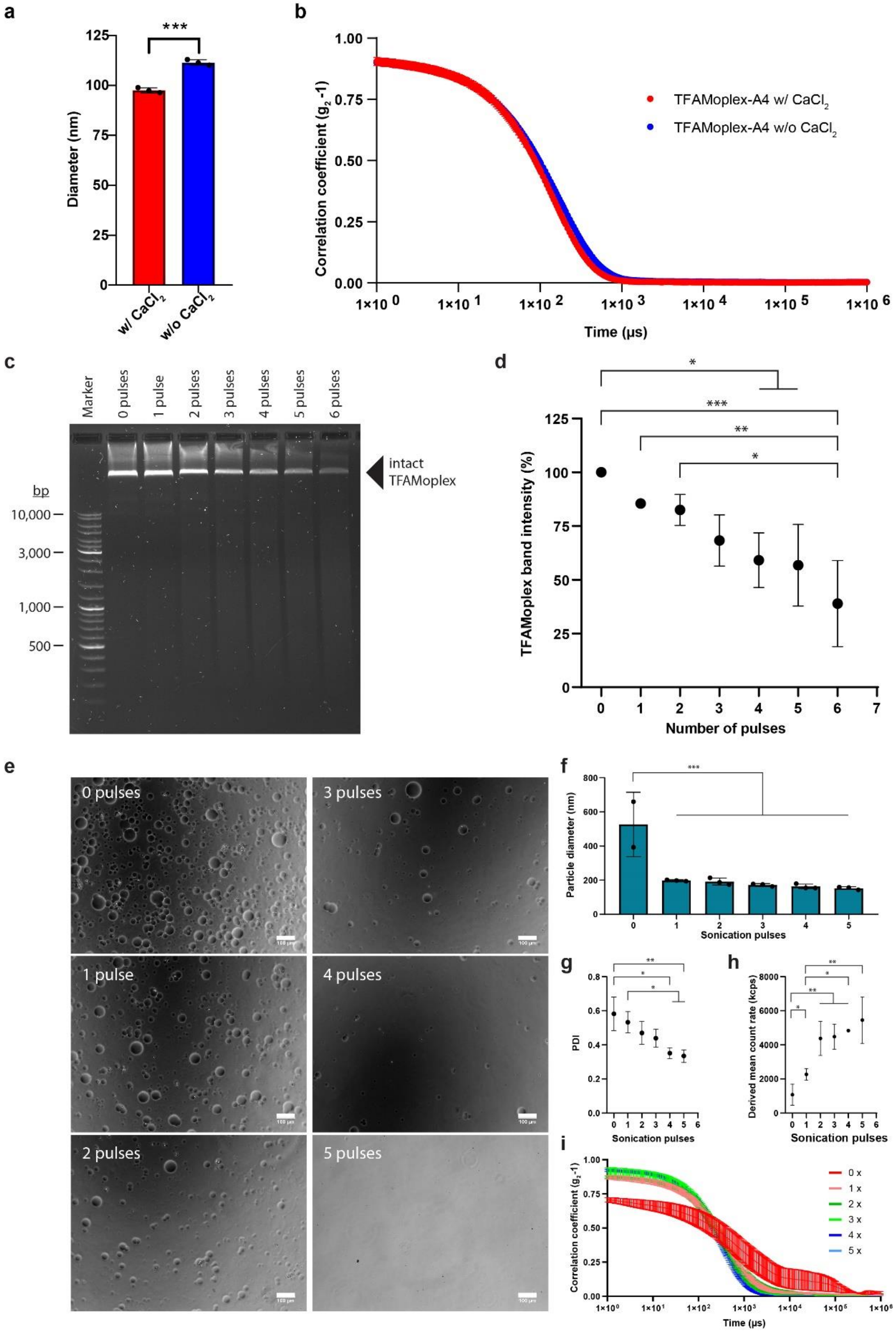

Supplementary Figure S3 – Size measurement of TFAMoplex-A4 as determined by DLS. a) Hydrodynamic diameter of TFAMplex-A4 in complex with pDNA. The TFAMoplexes were formed, followed by the addition of $CaCl_2$ to reach a final concentration of 1 mM (red) or a corresponding volume of Ca^{2+}-free buffer (blue). The mean

diameter is shown from three independent experiments (dots), each performed in technical triplicates (mean ± SD, N=3). ***p<0.001. b) Correlation function corresponding to panel a. Embedded legend corresponds to panel a and b. c) Impact of sonication pulses on TFAMoplex-A4. TFAMoplex-A4 was subjected to increasing numbers of 1-s sonication pulses (amplitude 1). Samples were then loaded onto a 0.8% agarose gel and DNA was stained with GelRed. A representative gel image is displayed. d) Densitometric quantification of the relative TFAMoplex band intensity from panel a. (Mean ± SD, N=3). *p<0.05, **p<0.01, ***p<0.001. e) Transformation of GUVs into small vesicles by sonication. e) Microscopy images showing anionic GUVs subjected to increasing numbers of 1-s sonication pulses. Scale bar = 100 µm. f-i) DLS analysis of GUVs before and after increasing numbers of 1-s sonication pulses, assessing the hydrodynamic diameter (f), PDI (g), particle count (h), and correlation function (i). Data in panels b-e represent mean values ± SD from three independent experiments, each performed in triplicates. *p<0.05, **p<0.01, ***p<0.001. Note: For non-sonicated GUVs, the particle size measurements exceeded the upper size limit of the DLS instrument and should be interpreted with caution. This supplementary figure is related to Figure 3.

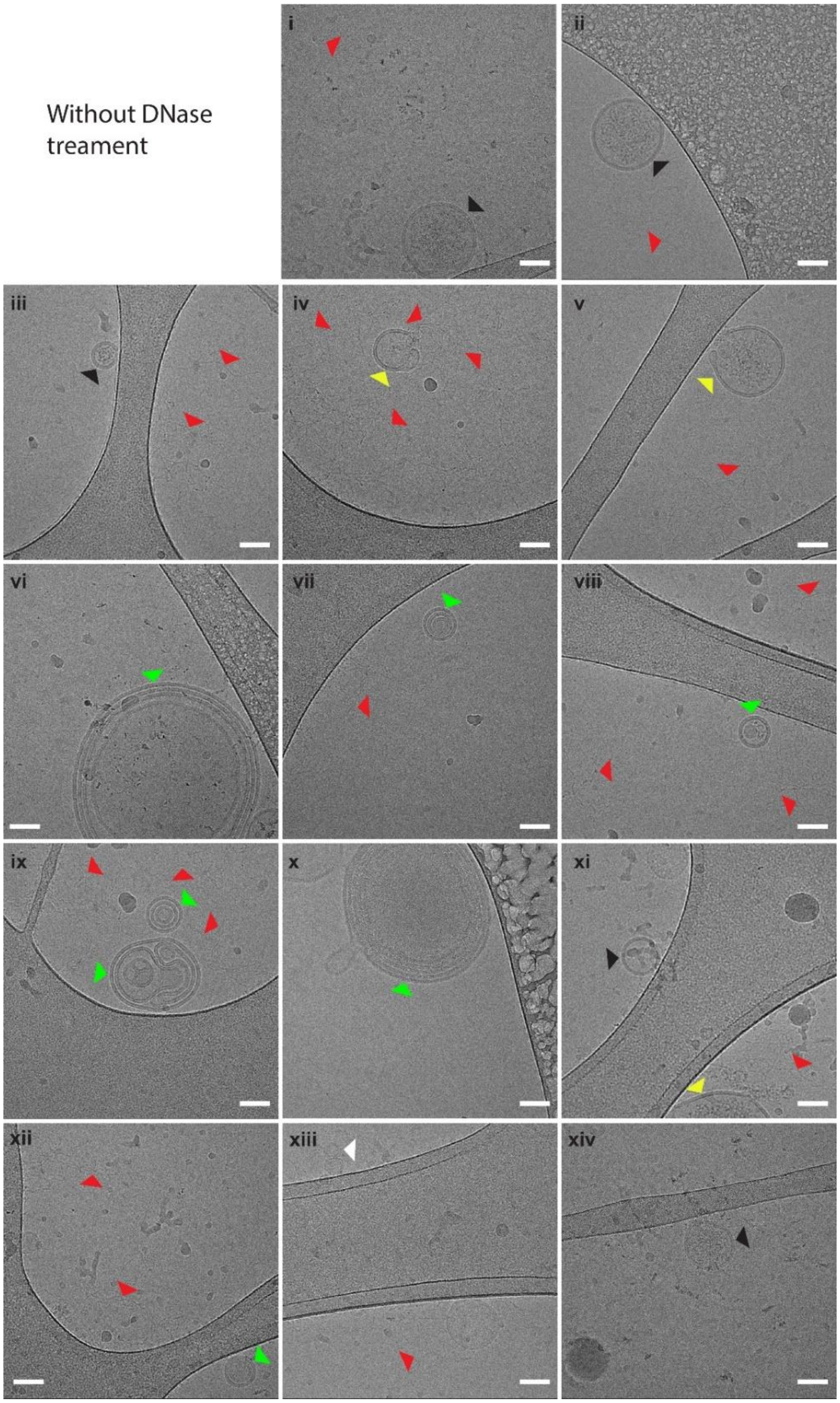

Supplementary Figure S4 – Additional cryo-TEM images of TFAMoplex-A4-loaded vesicles prepared without DNase treatment. Scale bars = 50 nm. i - iii) Intact filled vesicle, with free DNA. iv + v) Damaged vesicle, with free DNA. vi) Multilamellar vesicle. vii-ix) Multilamellar vesicle, with free DNA. x) Multilamellar and unilamellar vesicles. xi) Intact SUV, large damaged, filled unilamellar vesicle, free DNA. xii) Small multilamellar vesicle, with free DNA. xiii) small vesicles, with free DNA. xiv) Intact filled vesicle. Black arrowheads indicate ‘filled’ vesicles, yellow arrowheads ‘damaged’ vesicles, green arrowheads ‘multilamellar’ vesicles and red arrowheads show free DNA. Related to Figure 3.

With DNase treatment

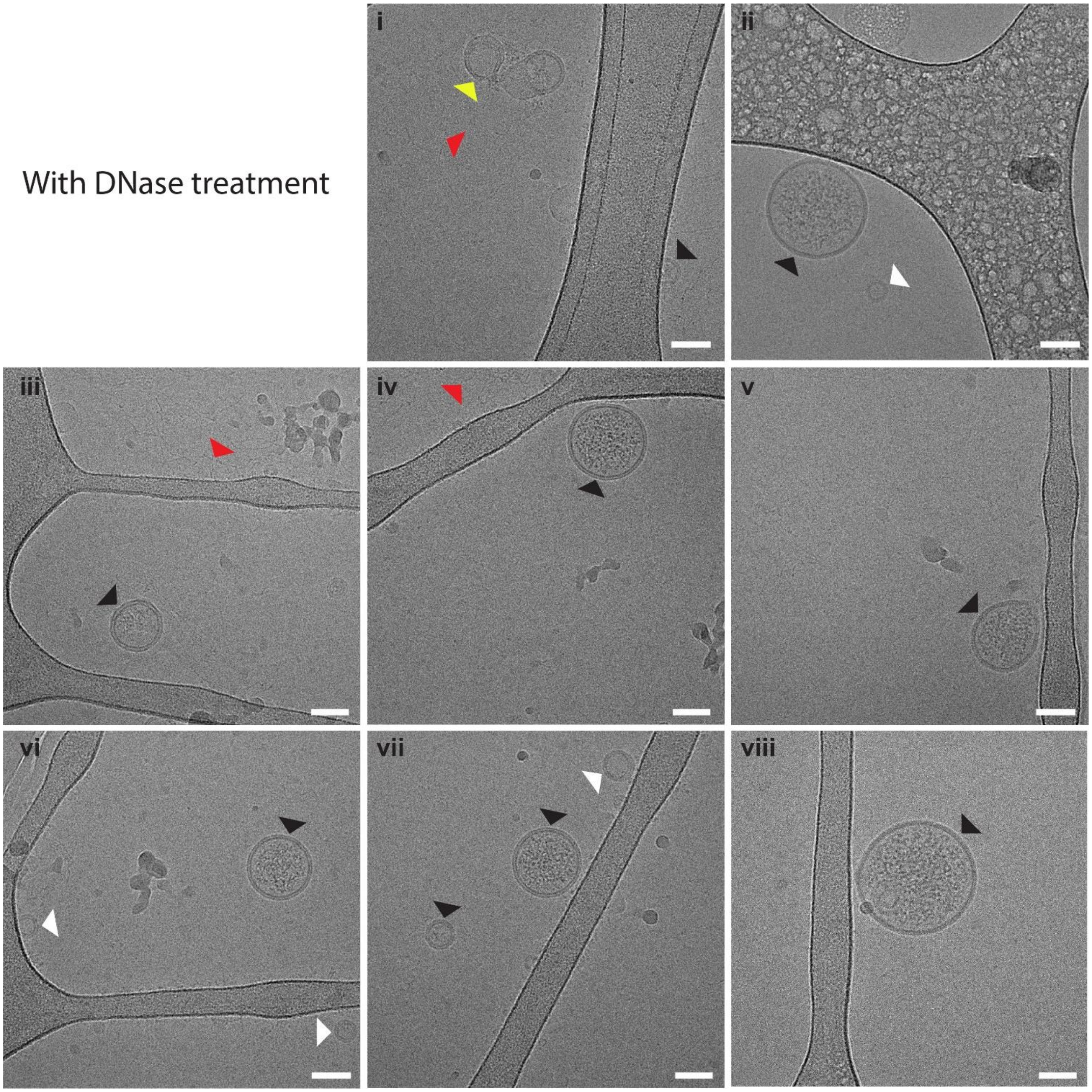

Supplementary Figure S5 – Additional cryo-TEM images of TFAMoplex-A4-loaded vesicles treated with DNase. Scale bars = 50 nm. i) Intact and damaged small vesicles, with free DNA. ii) Intact unilamellar vesicle, filled. Intact small unilamellar vesicle, empty. iii + iv) Intact unilamellar vesicles, filled, with free DNA. v) Unilamellar vesicle, filled, unclear if intact or damaged. vi) Intact unilamellar vesicle, filled. Intact SUVs, empty. vii) Intact unilamellar vesicles, one filled, one empty, one unclear. viii) Intact unilamellar vesicle, filled. Black arrowheads indicate 'filled' vesicles, white arrowheads 'empty' vesicles, yellow arrowheads 'damaged' vesicles and red arrowheads highlight free DNA. Related to Figure 3.

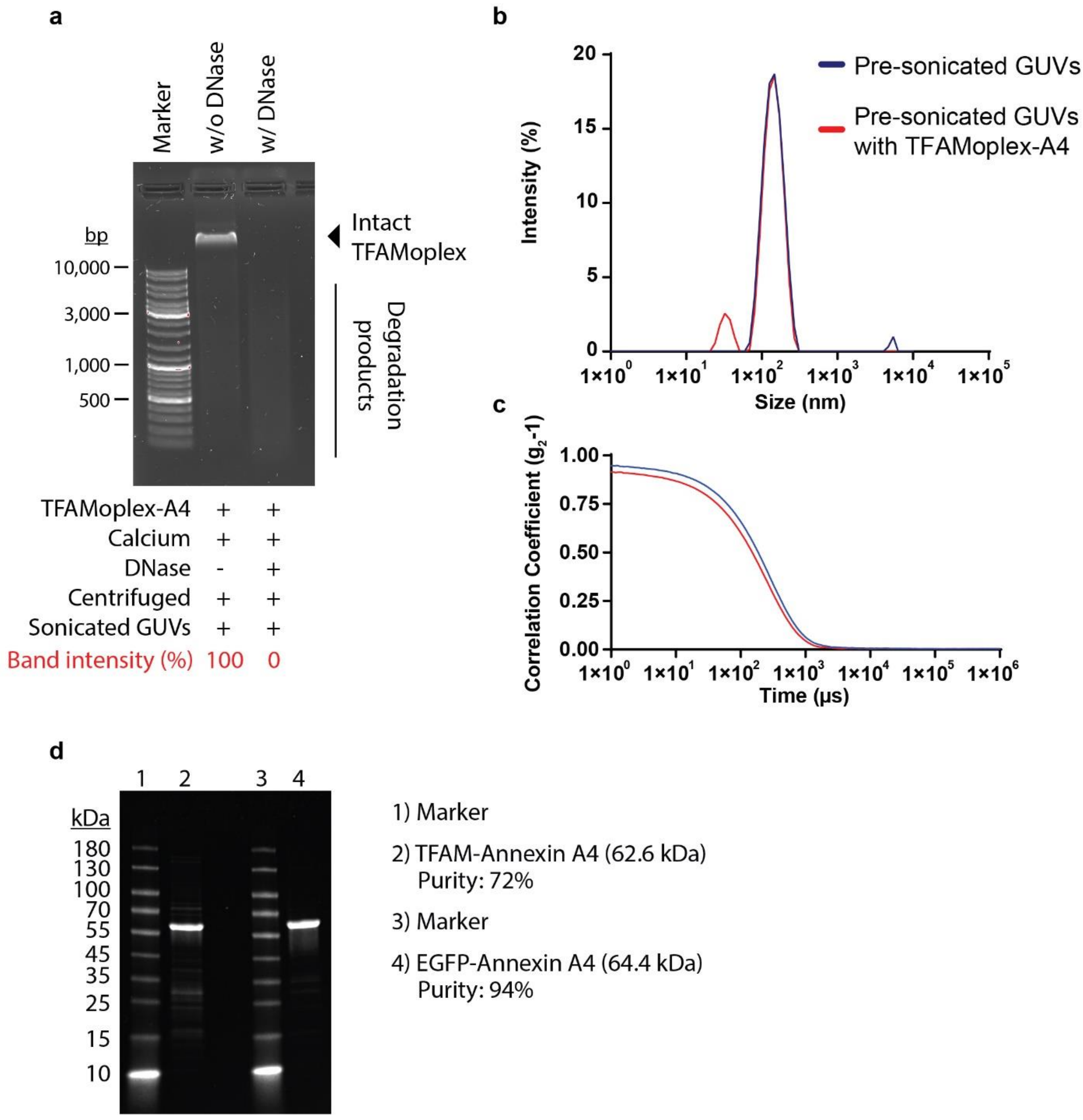

Supplementary Figure S6 – TFAMoplex recruitment and incorporation into LUVs (pre-sonicated anionic GUVs). a) To test whether TFAMoplex incorporation can also be achieved starting from LUVs instead of GUVs, the TFAMoplex-A4 was added to the exterior of pre-sonicated anionic GUVs in the presence of 1 mM $CaCl_2$. The samples were subsequently sonicated again (3 x 1-s pulses, amplitude 1) and treated with DNase to degrade non-encapsulated DNA. Shown is a 0.8% agarose gel with GelRed-stained DNA. b) DLS size analysis of pre-sonicated GUVs before addition of TFAMoplex-A4 (blue) and after addition of TFAMoplex-A4 and additional sonication (red). Displayed are the mean values of technical triplicates. c) Correlation curves corresponding to panel b. Related to Figure 4. d) SDS-PAGE analysis of TFAM-Annexin A4 T7D and EGFP-Annexin A4 T7D. SDS-PAGE was used to analyze the purity and molecular weights of the TFAM-Annexin A4 and EGFP-Annexin A4 constructs. The theoretical molecular weights and the purity of the constructs, as determined by densitometry analysis, are shown in the figure. Related to STAR Methods.

Supplementary Table S1 – DNA and protein sequences of recombinant TFAM-Annexin A4 T7D and EGFP-Annexin T7D.

**TFAM (amino acid 43-246) - Annexin A4 (amino acid 2-319, T7D)**	
DNA sequence	Protein sequence (His_6-TFAM-Annexin A4 T7D)
ATGGGTAGCAGCCATCATCATCATCACCACTC<br>TGCAGGAGGTACCGGACAAGCTTtGatgtcatctgtctt<br>gGCAAGTTGTCCAAAGAAACCTGTAAGTTCTTA<br>CCTTCGATTTTCTAAAGAACAACTACCCATATT<br>TAAAGCTCAGAACCCAGATGCAAAAACTACAG<br>AACTAATTAGAAGAATTGCCCAGCGTTGGAGG<br>GAACTTCCTGATTCAAAGAAAAAAATATATCA<br>AGATGCTTATAGGGCGGAGTGGCAGGTATATA<br>AAGAAGAGATAAGCAGATTTAAAGAACAGCT<br>AACTCCAAGTCAGATTATGTCTTTGGAAAAAG<br>AAATCATGGACAAACATTTAAAAAGGAAAGCT<br>ATGACAAAAAAAAAAAGAGTTAACACTGCTTGG<br>AAAACCAAAAAGACCTCGTTCAGCTTATAACG<br>TTTATGTAGCTGAAAGATTCCAAGAAGCTAAG<br>GGTGATTCACCGCAGGAAAAGCTGAAGACTGT<br>AAAGGAAAACTGGAAAAATCTGTCTGACTCTG<br>AAAAGGAATTATATATTCAGCATGCTAAAGAG<br>GACGAAACTCGTTATCATAATGAAATGAAGTC<br>TTGGGAAGAACAAATGATTGAAGTTGGACGAA<br>AGGATCTTCTACGTCGCACAATAAAGAAACAA<br>CGAAAATATGGTGCTGAGGAGTGTgGATCCGG<br>CGGTTCTGCAACTAAGGGAGGTGATGTTAAAG<br>CGGCCAGTGGGTTCAACGCGATGGAGGATGCG<br>CAGACATTACGCAAAGCGATGAAAGGACTGG<br>GCACGGACGAAGACGCGATCATCAGCGTTTTA<br>GCGTACCGCAATACTGCCCAACGCCAGGAGAT<br>TCGGACTGCATACAAGAGTACTATCGGTCGAG<br>ATTTAATTGACGATTTAAAGTCGGAGCTGTCA<br>GGTAACTTTGAGCAGGTGATTGTCGGTATGAT<br>GACACCTACGGTTTTATACGACGTGCAGGAAC<br>TCAGGCGTGCCATGAAGGGTGCAGGTACAGAC<br>GAAGGATGTCTAATTGAGATTCTGGCTTCACG<br>TACACCGGAGGAAATCAGGCGGATCAGTCAGA<br>CATACCAACAACAGTATGGTCGAAGTCTTGAA<br>GATGACATTCGCTCAGACACCAGCTTTATGTTT<br>CAGCGGGTATTGGTCAGCCTGTCAGCCGGAGG<br>TCGTGATGAAGGTAATTATCTTGACGACGCAC<br>TGGTGCGTCAGGATGCTCAAGATTTATATGAG<br>GCTGGAGAAAAGAAATGGGGTACCGACGAAG<br>TCAAATTTCTCACTGTACTCTGTTCTCGTAATC<br>GCAATCACTTGCTTCACGTTTTTGATGAGTACA<br>AACGTATCTCCCAGAAAGACATCGAACAATCT<br>ATTAAGTCTGAGACCTCGGGCTCATTTGAAGA<br>TGCCCTGCTCGCGATTGTCAAATGTATGCGCA<br>ATAAAAGCGCGTATTTTGCAGAGAAACTGTAC<br>AAGAGTATGAAAGGACTAGGCACAGACGACA<br>ATACATTGATTAGAGTGATGGTAAGTCGGGCA<br>GAAATAGACATGCTCGATATCCGCGCGCATTT<br>TAAGCGTCTGTATGGAAAGTCATTATATTCTTT<br>TATCAAAGGCGACACCTCTGGCGATTATCGTA	MGSSHHHHHHSAGGTGQALMSSVLASCPKKPVS<br>SYLRFSKEQLPIFKAQNPDAKTTELIRRIAQRWRE<br>LPDSKKKIYQDAYRAEWQVYKEEISRFKEQLTPS<br>QIMSLEKEIMDKHLKRKAMTKKKELTLLGKPKR<br>PRSAYNVYVAERFQEAKGDSPQEKLKTVKENW<br>KNLSDSEKELYIQHAKEDETRYHNEMKSWEEQ<br>MIEVGRKDLLRRTIKKQRKYGAEECGSGGSATK<br>GGDVKAASGFNAMEDAQTLRKAMKGLGTDED<br>AIISVLAYRNTAQRQEIRTAYKSTIGRDLIDDLKS<br>ELSGNFEQVIVGMMTPTVLYDVQELRRAMKGA<br>GTDEGCLIEILASRTPEEIRRISQTYQQQYGRSLED<br>DIRSDTSFMFQRVLVSLSAGGRDEGNYLDDALV<br>RQDAQDLYEAGEKKWGTDEVKFLTVLCSRNRN<br>HLLHVFDEYKRISQKDIEQSIKSETSGSFEDALLAI<br>VKCMRNKSAYFAEKLYKSMKGLGTDDNTLIRV<br>MVSRAEIDMLDIRAHFKRLYGKSLYSFIKGDTSG<br>DYRKVLLVLCGGDD

<table>
<tr><td>AGGTCTTGCTGGTTCTATGCGGGGGGGACGAT
TAA</td><td></td></tr>
<tr><td colspan="2">EGFP-Annexin A4 (amino acid 2-319, T7D)</td></tr>
<tr><td>DNA sequence</td><td>Protein sequence (His$_6$-EGFP-Annexin A4 T7D)</td></tr>
<tr><td>ATGGGTAGCAGCCATCATCATCATCACCACCA
AGCTTcgGTGAGCAAGGGCGAGGAGCTGTTCA
CCGGGGTGGTGCCCATCCTGGTCGAGCTGGAC
GGCGACGTAAACGGCCACAAGTTCAGCGTGTC
CGGCGAGGGCGAGGGCGATGCCACCTACGGC
AAGCTGACCCTGAAGTTCATCTGCACCACCGG
CAAGCTGCCCGTGCCCTGGCCCACCCTCGTGA
CCACCCTGACCTACGGCGTGCAGTGCTTCAGC
CGCTACCCCGACCACATGAAGCAGCACGACTT
CTTCAAGTCCGCCATGCCCGAAGGCTACGTCC
AGGAGCGCACCATCTTCTTCAAGGACGACGGC
AACTACAAGACCCGCGCCGAGGTGAAGTTCGA
GGGCGACACCCTGGTGAACCGCATCGAGCTGA
AGGGCATCGACTTCAAGGAGGACGGCAACATC
CTGGGGCACAAGCTGGAGTACAACTACAACAG
CCACAACGTCTATATCATGGCCGACAAGCAGA
AGAACGGCATCAAGGTGAACTTCAAGATCCGC
CACAACATCGAGGACGGCAGCGTGCAGCTCGC
CGACCACTACCAGCAGAACACCCCCATCGGCG
ACGGCCCCGTGCTGCTGCCCGACAACCACTAC
CTGAGCACCCAGTCCGCCCTGAGCAAAGACCC
CAACGAGAAGCGCGATCACATGGTCCTGCTGG
AGTTCGTGACCGCCGCCGGGATCACTCTCGGC
ATGGACGAGCTGTACAAGgGATCCGGCGGTTC
TGCAACTAAGGGAGGTGATGTTAAAGCGGCCA
GTGGGTTCAACGCGATGGAGGATGCGCAGACA
TTACGCAAAGCGATGAAAGGACTGGGCACGG
ACGAAGACGCGATCATCAGCGTTTTAGCGTAC
CGCAATACTGCCCAACGCCAGGAGATTCGGAC
TGCATACAAGAGTACTATCGGTCGAGATTTAA
TTGACGATTTAAAGTCGGAGCTGTCAGGTAAC
TTTGAGCAGGTGATTGTCGGTATGATGACACC
TACGGTTTTATACGACGTGCAGGAACTCAGGC
GTGCCATGAAGGGTGCAGGTACAGACGAAGG
ATGTCTAATTGAGATTCTGGCTTCACGTACACC
GGAGGAAATCAGGCGGATCAGTCAGACATACC
AACAACAGTATGGTCGAAGTCTTGAAGATGAC
ATTCGCTCAGACACCAGCTTTATGTTTCAGCGG
GTATTGGTCAGCCTGTCAGCCGGAGGTCGTGA
TGAAGGTAATTATCTTGACGACGCACTGGTGC
GTCAGGATGCTCAAGATTTATATGAGGCTGGA
GAAAAGAAATGGGGTACCGACGAAGTCAAAT
TTCTCACTGTACTCTGTTCTCGTAATCGCAATC
ACTTGCTTCACGTTTTTGATGAGTACAAACGTA
TCTCCCAGAAAGACATCGAACAATCTATTAAG
TCTGAGACCTCGGGCTCATTTGAAGATGCCCT
GCTCGCGATTGTCAAATGTATGCGCAATAAAA
GCGCGTATTTTGCAGAGAAACTGTACAAGAGT
ATGAAAGGACTAGGCACAGACGACAATACATT
GATTAGAGTGATGGTAAGTCGGGCAGAAATAG
ACATGCTCGATATCCGCGCGCATTTTAAGCGTC
TGTATGGAAAGTCATTATATTCTTTTATCAAAG</td><td>MGSSHHHHHHQASVSKGEELFTGVVPILVELDG
DVNGHKFSVSGEGEGDATYGKLTLKFICTTGKLP
VPWPTLVTTLTYGVQCFSRYPDHMKQHDFFKSA
MPEGYVQERTIFFKDDGNYKTRAEVKFEGDTLV
NRIELKGIDFKEDGNILGHKLEYNYNSHNVYIMA
DKQKNGIKVNFKIRHNIEDGSVQLADHYQQNTPI
GDGPVLLPDNHYLSTQSALSKDPNEKRDHMVLL
EFVTAAGITLGMDELYKGSGGSATKGGDVKAAS
GFNAMEDAQTLRKAMKGLGTDEDAIISVLAYRN
TAQRQEIRTAYKSTIGRDLIDDLKSELSGNFEQVI
VGMMTPTVLYDVQELRRAMKGAGTDEGCLIEIL
ASRTPEEIRRISQTYQQQYGRSLEDDIRSDTSFMF
QRVLVSLSAGGRDEGNYLDDALVRQDAQDLYE
AGEKKWGTDEVKFLTVLCSRNRNHLLHVFDEY
KRISQKDIEQSIKSETSGSFEDALLAIVKCMRNKS
AYFAEKLYKSMKGLGTDDNTLIRVMVSRAEIDM
LDIRAHFKRLYGKSLYSFIKGDTSGDYRKVLLVL
CGGDD</td></tr>
</table>

GCGACACCTCTGGCGATTATCGTAAGGTCTTG CTGGTTCTATGCGGGGGGGACGATTAA	
